# *MicroRNA-449a* deficiency promotes colon carcinogenesis

**DOI:** 10.1038/s41598-017-10500-0

**Published:** 2017-09-06

**Authors:** Masanori Niki, Kohei Nakajima, Daichi Ishikawa, Jun Nishida, Chieko Ishifune, Shin-ichi Tsukumo, Mitsuo Shimada, Shinji Nagahiro, Yoshinori Mitamura, Koji Yasutomo

**Affiliations:** 1 Department of Immunology & Parasitology, Graduate School of Medicine, Tokushima University, Tokushima, Japan; 2 Department of Ophthalmology, Graduate School of Medicine, Tokushima University, Tokushima, Japan; 3 Department of Neurosurgery, Graduate School of Medicine, Tokushima University, Tokushima, Japan; 4 Department of Surgery, Graduate School of Medicine, Tokushima University, Tokushima, Japan

## Abstract

MicroRNAs have broad roles in tumorigenesis and cell differentiation through regulation of target genes. Notch signaling also controls cell differentiation and tumorigenesis. However, the mechanisms through which Notch mediates microRNA expression are still unclear. In this study, we aimed to identify microRNAs regulated by Notch signaling. Our analysis found that microRNA-449a (*miR-449a*) was indirectly regulated by Notch signaling. Although *miR-449a*-deficient mice did not show any Notch-dependent defects in immune cell development, treatment of *miR-449a*-deficient mice with azoxymethane (AOM) or dextran sodium sulfate (DSS) increased the numbers and sizes of colon tumors. These effects were associated with an increase in intestinal epithelial cell proliferation following AOM/DSS treatment. In patients with colon cancer, *miR-449a* expression was inversely correlated with disease-free survival and histological scores and was positively correlated with the expression of *MLH1* for which loss-of function mutations have been shown to be involved in colon cancer. Colon tissues of *miR-449a*-deficient mice showed reduced *Mlh1* expression compared with those of wild-type mice. Thus, these data suggested that *miR-449a* acted as a key regulator of colon tumorigenesis by controlling the proliferation of intestinal epithelial cells. Additionally, activation of *miR-449a* may represent an effective therapeutic strategy and prognostic marker in colon cancer.

## Introduction

Mature microRNAs (miRNAs) are noncoding RNAs of approximately 22 nucleotides that regulate gene expression by targeting the 3′-untranslated regions (UTRs) of mRNAs, resulting in inhibition of mRNA translation^1, 2^. MiRNAs regulate various aspects of cell physiology, including proliferation, differentiation, cell death, and development^1^. Moreover, aberrantly expressed miRNAs are involved in tumorigenesis, as either oncogenes or tumor suppressors^3–5^. For example, *miR-34a-5p* suppresses colorectal cancer metastasis, and its expression predicts recurrence in patients with stage II/III colorectal cancer^6^. Additionally, *miR-183* functions as an oncogene and promotes tumor cell migration^7^. Therefore, miRNAs may be potential targets for cancer therapy.

Notch signaling also has pleiotropic roles in cell differentiation, proliferation, and cell death, and dysregulation of Notch is involved in many types of malignant tumors, including T-cell acute lymphoblastic leukemia (T-ALL) and lymphoma^8–10^. Notably, we have previously reported that Notch is an essential mediator of T-cell differentiation^11, 12^ and maintenance of memory CD4 T cells^13^. Moreover, in studies of Notch and cancer, frequent active mutations in Notch1 have been reported in patients with T-ALL^14, 15^ and gain-of-function mutations and copy numbers of Notch2 are increased in diffuse large B-cell lymphoma^16^.

*MicroRNA-449a* (*miR-449a*) is a member of the *miR-449* family (*miR-449a*, *miR-449b*, and *miR-449c*). The *miR-449* cluster contains sequences and secondary structures similar to those of the *miR-34* family and has therefore been classified as a single family of miRNAs. The expression of *miR-449a* is decreased in several cancers, including gastric and bladder cancer^17, 18^. Furthermore, *miR-449a* regulated several genes associated with tumorigenesis, including the gene encoding histone deacetylase (HDAC)^19^ and CDC25A^20^, suggesting that *miR-449a* may have oncogenic effects. However, the roles of *miR-449a* in tumorigenesis *in vivo* have not yet been determined.

In this study, we investigated whether miRNAs were regulated by Notch signaling. Our results showed that *miR-449a* was upregulated by Notch signaling. Unexpectedly, *miR-449a*-deficient mice did not show any defects in the development of T cells, marginal zone B cells, and CD8α^−^ splenic dendritic cells, all of which are regulated by Notch signaling. Additionally, *miR-449a*-deficient mice showed increased susceptibility to azoxymethane (AOM) and dextran sodium sulfate (DSS)-induced colon cancer with increased proliferation of intestinal epithelial cells. Furthermore, the expression of *miR-449a* was inversely correlated with histological scores and disease-free survival in patients with colon cancer. Deficiency of *miR-449a* in the colon resulted in downregulation of *Mlh1*, and expression of *miR-449a* was positively correlated with that of *Mlh1* in patients with colon cancer. These data highlighted the role of *miR-449a* as a tumor suppressor in colon cancer and suggested that *miR-449a* may be a therapeutic target in the treatment of colon cancer.

## Results

### Notch regulated miR-449a expression

**Figure 1:**
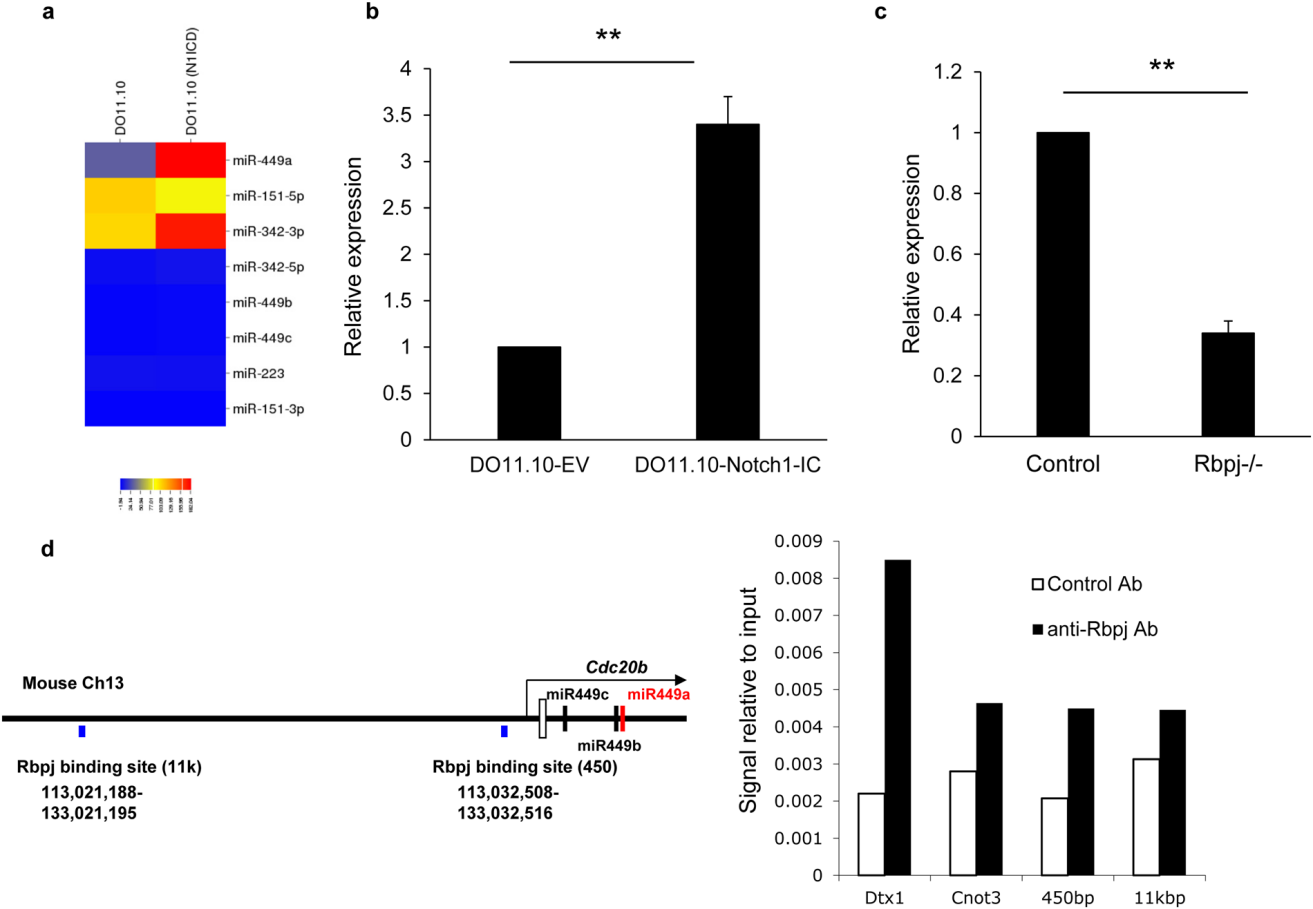
Notch controlled *miR-449a* expression. (**a**) Microarray analysis of miRNAs in DO11.10 cells infected with control retrovirus (DO11.10-EV) or retrovirus encoding the intracellular domain of Notch1 (DO11.10-Notc1-IC). (**b**) The expression of *miR-449a* in DO11.10 cells infected with control retrovirus (DO11.10-EV) or retrovirus encoding the intracellular domain of Notch1 (DO11.10-Notch1-IC), as analyzed by real-time PCR. Expression relative to that of snoRNA202 is shown. The data are shown as means ± SDs. **p < 0.01. (**c**) The expression of *miR-449a* in T cells from C57BL/6 mice crossed with CD4-Cre transgenic mice (control) or Rbpj^flox/flox^ mice crossed with CD4-Cre transgenic (Rbpj^−/−^) mice,as analyzed by real-time PCR. Expression relative to that of snoRNA202 is shown. The data are shown as means ± SDs. **p < 0.01. (**d**) Diagram of the mouse *miR-449a* locus (left). Two putative Rbpj binding regions (450 and 11 K) are shown. Chromatin immunoprecipitation assays were performed using anti-Rbpj antibodies, and the precipitated DNA was amplified by primers for *Dtx1* and two putative Rbpj binding regions (right). As a negative control, primers for *Cnot3* were used. The relative value was calculated using the value of anti-Rbpj antibody/input. The data are representative of three independent experiments.

We first searched for miRNAs regulated by Notch signaling using an miRNA microarray. Because the interactions between Notch and Notch ligands allow γ-secretase to cleave Notch, resulting in translocation of the intracellular domain of Notch into the nucleus^9^, overexpression of the intracellular domain of Notch in cells can activate Notch signaling. We compared the expression of miRNA between DO11.10 cells infected with control retrovirus or retrovirus carrying the intracellular domain of Notch1 (Fig. 1a). Only *miR-449a* was upregulated by more than 3 fold in DO.11.10 cells infected with the intracellular domain of Notch1, as confirmed by real-time polymerase chain reaction (PCR) analysis (Fig. 1a and b). Additionally, *miR-223* ^21^, which has been reported to be related to Notch signaling, was not altered in this comparison (Fig. 1a). Because Rbpj is essential for Notch signaling, we compared the expression of *miR-449a* in Rbpj-deficient and wild-type T cells. Rbpj-deficient T cells from Rbpj^flox/flox^ crossed with CD4-Cre transgenic mice showed substantially reduced expression of *miR-449a* compared with that in wild-type cells (Fig. 1c), although *miR-449a* expression was still detected in Rbpj-deficient cells. These data demonstrated that Notch was an upstream regulator of *miR-449a*.

We next sought to evaluate whether Notch signaling directly controlled *miR-449a* expression. There were two putative Rbpj binding regions upstream of the *miR-449a* locus in homologous regions shared between mice and humans (depicted as 11k and 450) (Fig. 1d). We performed chromatin immunoprecipitation assays with anti-Rbpj antibodies and detected binding of Rbpj in *Dtx1*, a known Notch target gene. The relative increase in PCR products in two regions by anti-Rbpj antibody compared with that of the control antibody was similar to that of the *Cnot3* region (Fig. 1d), suggesting the indirect regulation of *miR-449a* expression by Notch signaling.

### Establishment of miR-449a-deficient mice

**Figure 2:**
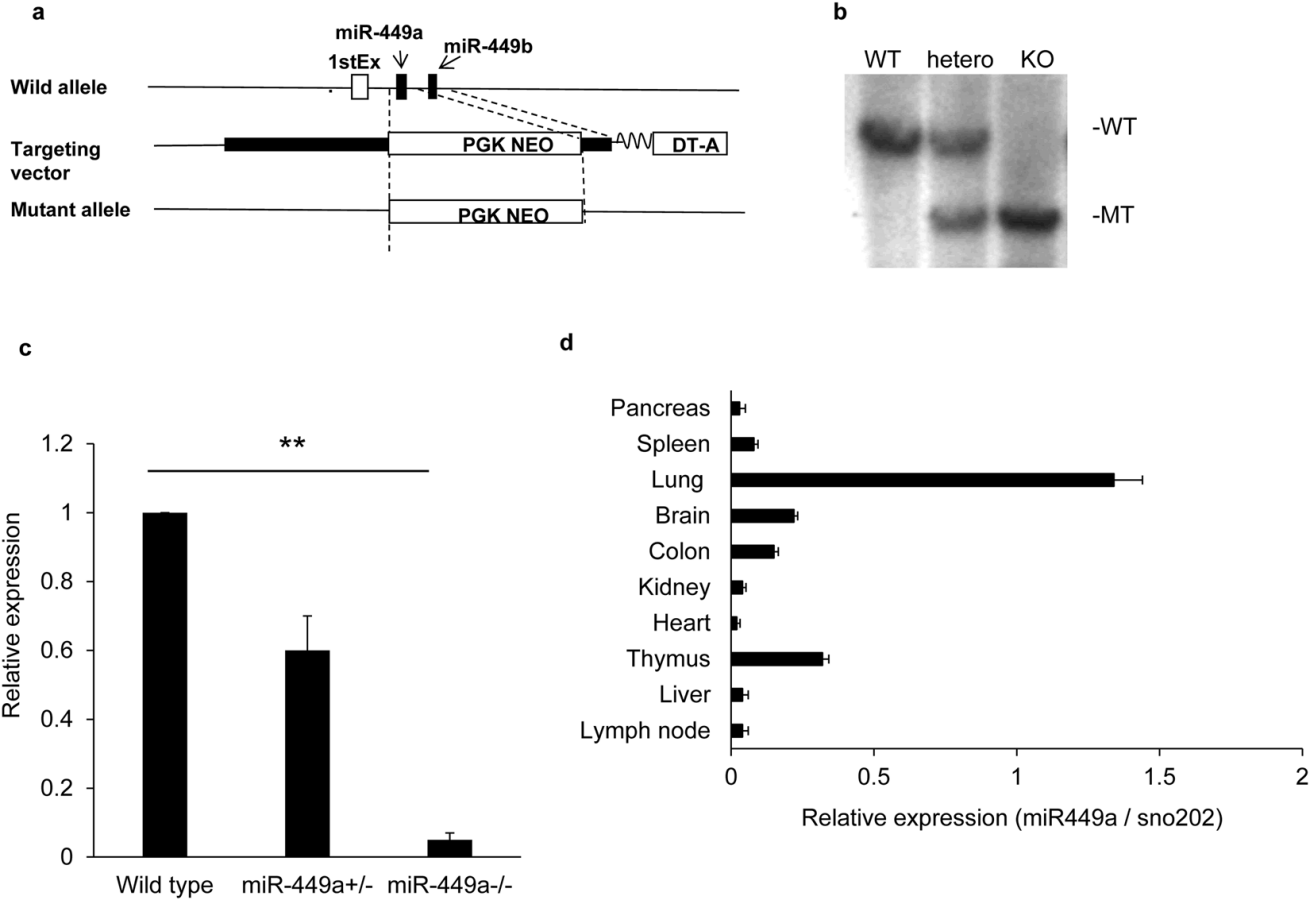
Establishment of *miR-449a*-deficient mice. (**a**) Configuration of the targeting vector of *miR-449a*-deficient mice. (**b**) Genomic DNA was digested with Bcl1 and subjected to Southern blot analysis. Genomic DNA from wild-type or *miR-449a* ^−/−^ mice was amplified by PCR primers that detected the wild-type or mutant allele. (**c**) RNA was isolated from T cells of wild-type, *miR-449a* ^+/+^, and *miR-449a* ^−/−^ mice, and the expression of *miR-449a* was evaluated by real-time PCR. Expression relative to that of *G3pdh* is shown. The data are shown as means ± SDs. **p < 0.01. (**d**) Total RNA was isolated from the indicated organs, and the expression of *miR-449a* in each organ was evaluated by real-time PCR. Expression relative to *G3pdh* is shown. The data are representative of three independent experiments.

In order to evaluate the roles of *miR-449a* in immune cell development and tumorigenesis, we established *miR-449a*-deficient (*miR-449a* ^−/−^) mice. The *miR-449a* locus was replaced with a neo-cassette (Fig. 2a), and homologous recombination was confirmed by Southern blotting and PCR (Fig. 2b and Supplementary Figure 2). T cells from *miR-449a* ^+/+^ or *miR-449a* ^−/−^ mice showed approximately 50% expression or no expression of *miR-449a*, respectively, compared with those of wild-type cells (Fig. 2c), indicating that complete deficiency of *miR-449a* expression was achieved in *miR-449a* ^−/−^ mice. *miR-449a* ^−/−^ mice were born according to Mendelian inheritance rules and did not show any gross body changes. *miR-449a* ^−/−^ mice were viable for up to at least 60 weeks of age (data not shown).

We assessed the expression of *miR-449a* in various organs by real-time PCR. Our results showed that *miR-449a* was highly expressed in the thymus and lung, but was expressed at low levels in the brain and colon (Fig. 2d).

### MiR-449a deficiency did not affect immune cell developmen*t*

**Figure 3:**
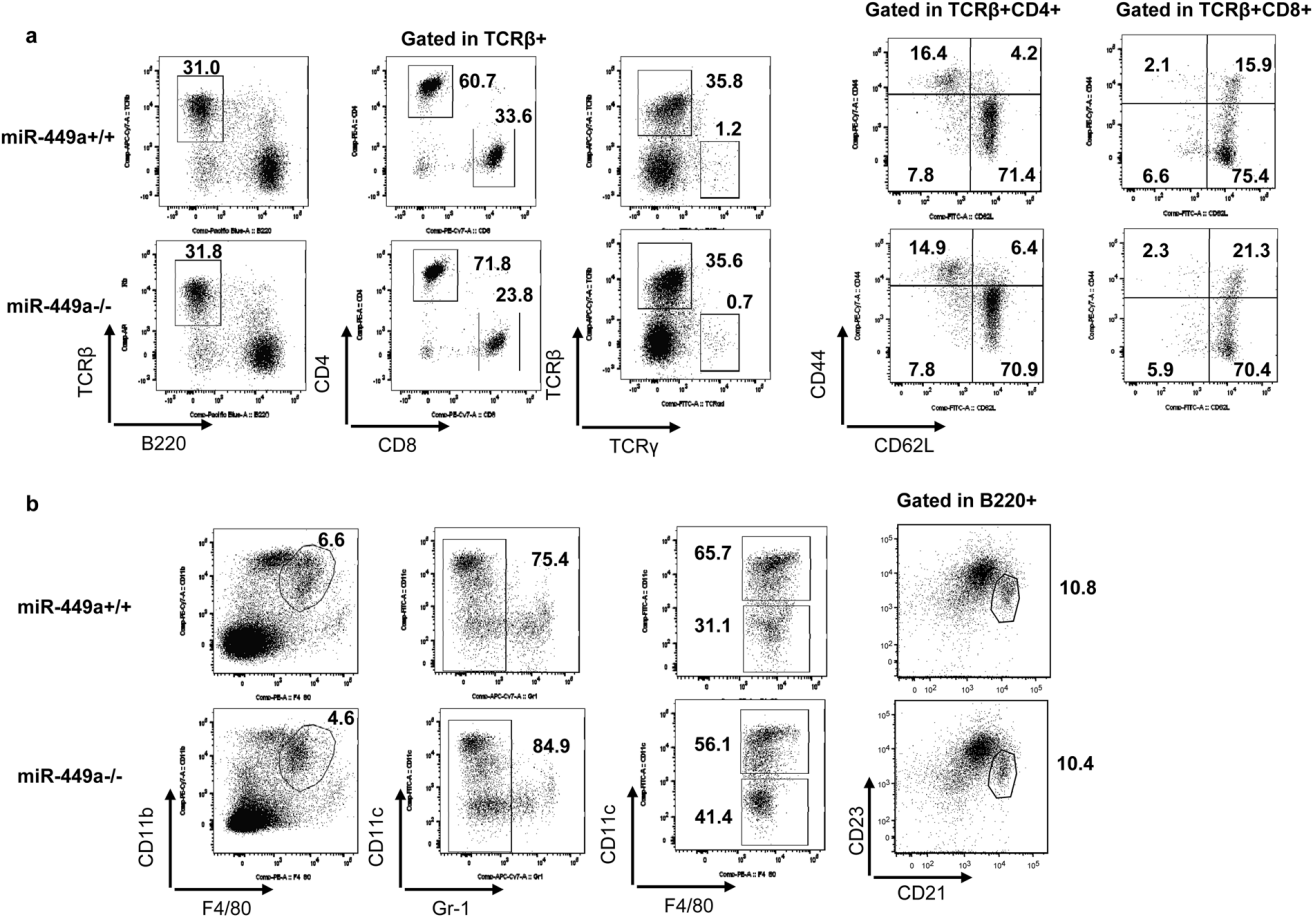
Unimpaired development of immune cells in *miR-449a* ^−/−^ mice. Single-cell suspensions derived from the spleens of wild-type and *miR-449a* ^−/−^ mice were stained with (**a**) anti-CD4, anti-CD8, anti-TCRβ, anti-TCRγ, anti-B220, anti-CD44, and anti-CD62L antibodies or (**b**) anti-CD11b, anti-F4/80, anti-Gr1, anti-CD11c, anti-B220, anti-CD21, and anti-CD23 antibodies, and the expression levels of the targets were evaluated by flow cytometry. The numbers indicate the percentages for each section.

Because Notch signaling regulates early T-cell development and effector T-cell differentiation^9, 22^, we evaluated changes in immune cell numbers in *miR-449a* ^−/−^ mice. The total cell numbers in the thymus, lymph nodes, and spleen were comparable between wild-type and *miR-449a* ^−/−^ mice (Fig. 3a). The ratios of CD4 to CD8 T cells in the thymus, TCRβ^+^ to TCRγ^+^ T cells in the spleen and lymph nodes, TCRβ to B220 cells in the spleen and lymph nodes, and CD44 to CD62L expression in CD4 or CD8 T cells in the spleen were also comparable between wild-type and *miR-449a* ^−/−^ mice (Fig. 3a). Notch signaling controls the survival of CD8α^−^ dendritic cells and the development of marginal zone B cells in the spleen^9^. Notably, the frequencies of CD8α^−^ dendritic cells and marginal zone B cells (CD21^+^CD23^+^) in *miR-449a* ^−/−^ mice were equivalent to those of wild-type mice (Fig. 3b).Th1, Th2, and Th17 cell differentiation in *miR-449a* ^−/−^ mice was equivalent to that in control mice (Supplementary Figure 1). These data suggested that *miR-449a* deficiency did not affect immune cell development or differentiation, which are regulated by Notch signaling.

### Promotion of colon cancer after AOM/DSS treatment in miR-449a^−/−^ mice

**Figure 4:**
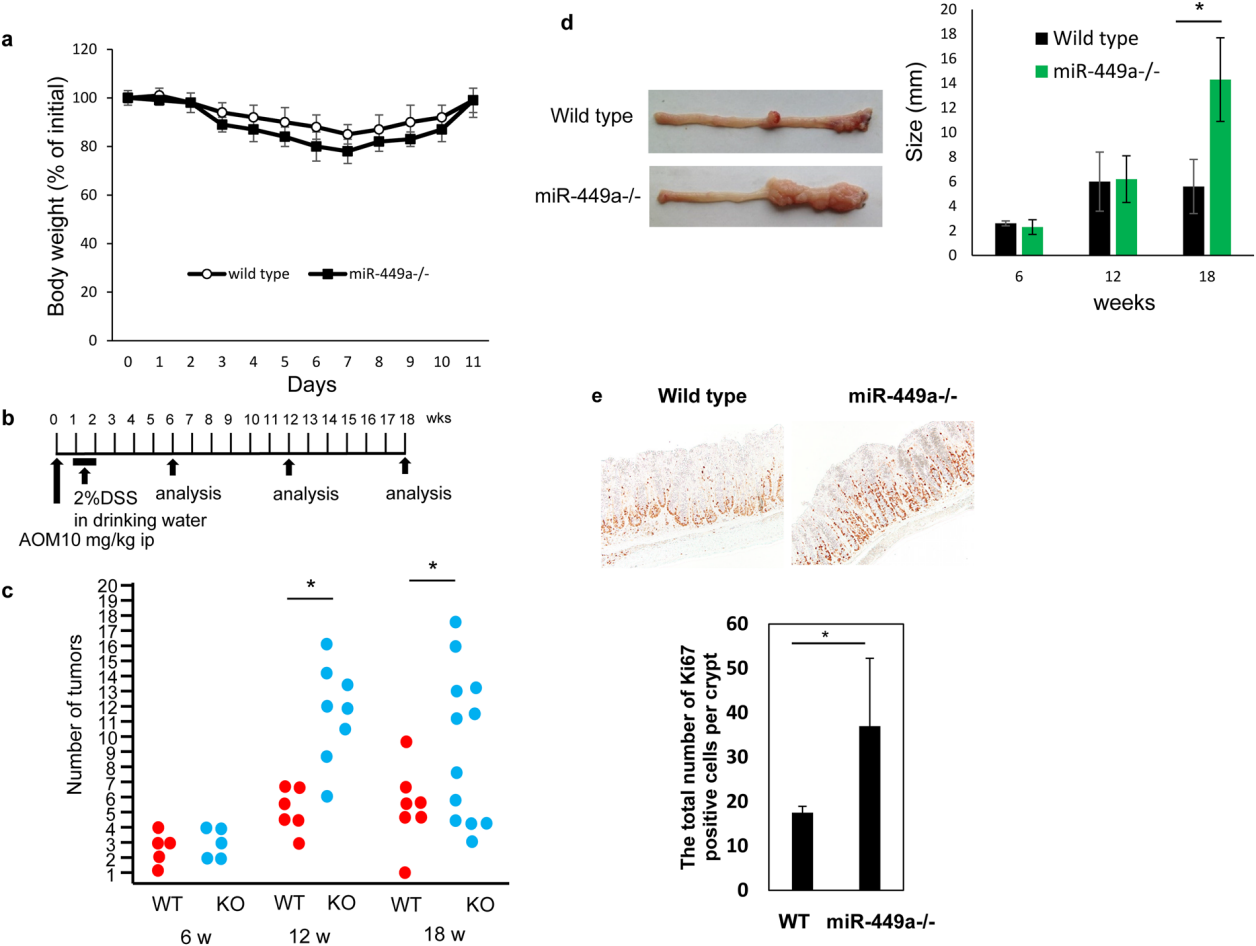
Increased colon tumor formation after AOM/DSS treatment in *miR-449a* ^−/−^ mice. (**a**) Wild-type and *miR-449a* ^−/−^ mice were treated with 2% DSS in drinking water for 7 days, and body weights were evaluated. The body weight before DSS treatment was set as 100%. The data are shown as means ± SDs. (**b**) Protocol of AOM and DSS treatment; wild-type and *miR-449a* ^−/−^ mice received 2% DSS through the drinking water for 7 days and were injected with AOM for 7 days beginning on day 0. (**c**) Number of colon tumors in each wild-type (WT; red circle) or *miR-449a* ^−/−^ (KO; blue circle) mouse were measured at 6, 12, and 18 weeks after AOM/DSS treatment. Photographs are representative colon tissues at 18 weeks after AOM/DSS treatment. The data are shown as means ± SDs. *p < 0.05. (**d**) Sizes of colon tumors in wild-type (black) or *miR-449a* ^−/−^ (green) mice were measured at 6, 12, and 18 weeks after AOM/DSS treatment. The data are shown as means ± SDs. *p < 0.05. (**e**) Wild-type (WT) and *miR-449a* ^−/−^ mice were treated with AOM and 2% DSS for 7 days, and Ki-67 staining of colonic tissue sections was performed on day 7 with anti-Ki-67 antibodies and counterstaining with hematoxylin. The number of Ki-67-positive cells in 10 villi was counted. The data are shown as means ± SDs. *p < 0.05. The data are representative of three independent experiments.

Because previous reports have shown that *miR-449a* is involved in tumorigenesis in prostate, breast, lung, and gastric cancers^23–26^ and because *miR-449a* is expressed in the colon, we next sought to assess the roles of *miR-449a* in the tumorigenesis of colon cancer. We first treated wild-type or *miR-449a* ^−/−^ mice with 2% DSS and monitored body weight loss. There were no significant differences in body weight between the two groups, although body weight loss in *miR-449a* ^−/−^ mice tended to be more abundant than that in wild-type mice (Fig. 4a). We then treated wild-type and *miR-449a* ^−/−^ mice with AOM and 2% DSS to induce colon cancer (Fig. 4b) and measured the numbers of mice with tumors, tumor numbers in each mouse, and sizes of tumors at 6, 12, and 18 weeks after AOM treatment. Tumor incidence was 100%, and polyp numbers were comparable in both experimental groups and controls (data not shown). Tumors were found in both wild-type and *miR-449a* ^−/−^ mice at 6 weeks after AOM treatment, and *miR-449a* ^−/−^ mice had significantly more tumors than wild-type mice at 12 and 18 weeks after AOM treatment (Fig. 4c). The sizes of tumors were also larger in *miR-449a* ^−/−^ mice at 18 weeks after AOM treatment, with approximately 50% of *miR-449a* ^−/−^ mice developing tumors larger than 12 mm in diameter (Fig. 4d).

To characterize the nature of the deregulated carcinogenic signals in *miR-449a* ^−/−^ mice, we examined colonic epithelial proliferation in the tumorigenic process in the intestine. Ki67 was mainly expressed in the basal region of the colon in wild-type and *miR-449a* ^−/−^ mice (Fig. 4e). The number of intestinal epithelial cells expressing Ki67 was much larger in *miR-449a* ^−/−^ mice than in wild-type mice (Fig. 4e), demonstrating the increased proliferation of intestinal epithelial cells in *miR-449a* ^−/−^ mice after AOM/DSS treatment. Taken together, these data suggested that *miR-449a* acted as a tumor suppressor in colon cancer.

### Disease-free survival in colon cancer was much longer in patients with higher miR-449a expression

**Figure 5:**
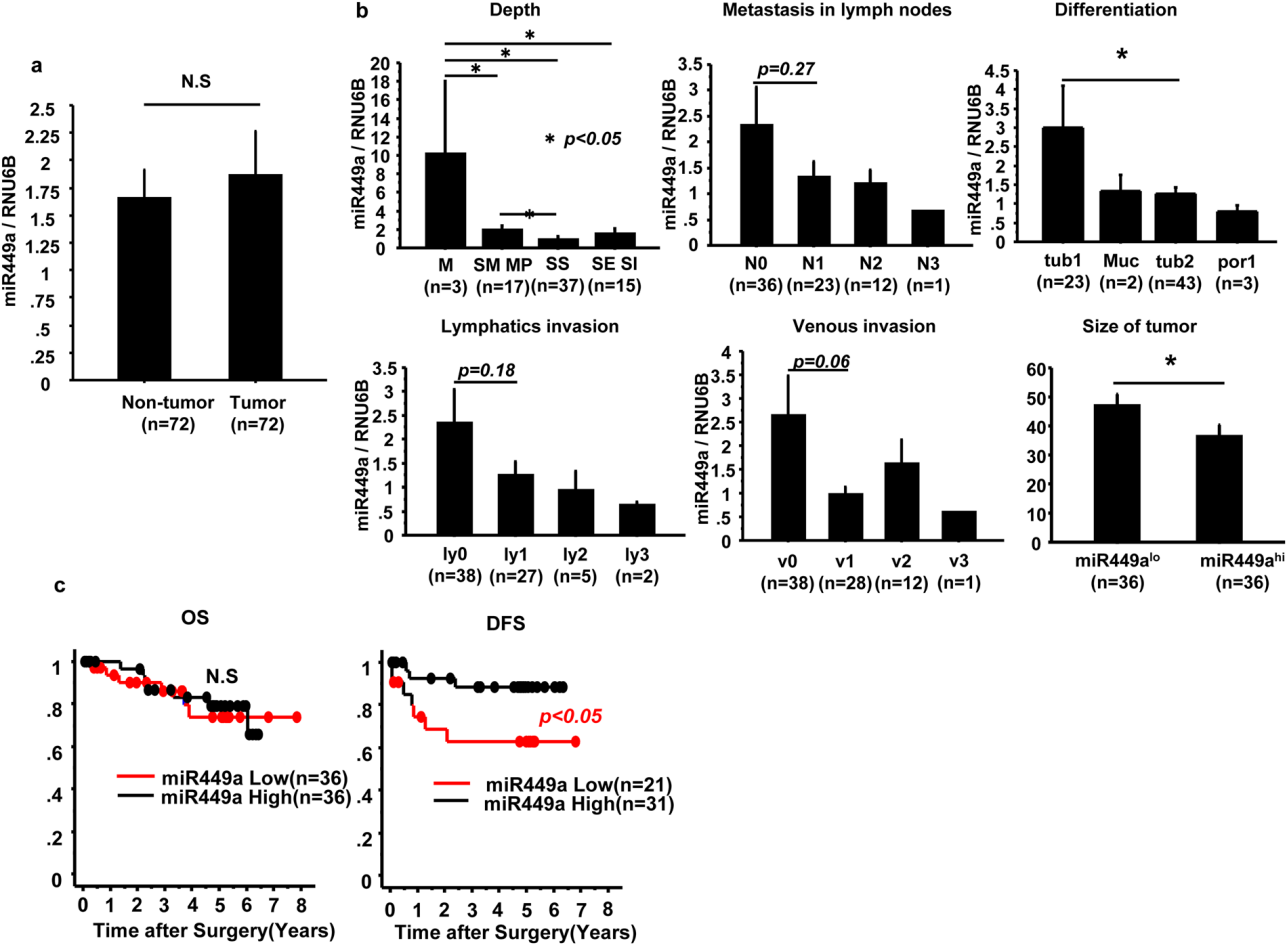
m*iR-449a* expression was inversely correlated with disease-free survival in patients with colon cancer. (**a**) The expression of *miR-449a* in noncancerous and cancerous tissues from patients with colon cancer was evaluated by real-time PCR. The data are shown as means ± SDs. n.s.: not significant. (**b**) *miR-449a* expression levels were evaluated based on the grade of colon cancer, which was determined according to depth, lymph nodes metastasis, differentiation, lymphatic invasion, venous invasion, and tumor size. The data are shown as means ± SDs. *P* values are shown for each comparison. (**c**) Overall survival and disease-free survival were evaluated between *miR-449a* ^low^ (red) and *miR-449a* ^hi^ (black) patients. The data are shown as means ± SDs. *p < 0.05. The data are representative of three independent experiments.

The results obtained in *miR-449a* ^−/−^ mice led us to look for a link between *miR-449a* expression and pathology or prognosis in patients with colon cancer. We compared the expression of *miR-449a* in the intact colon and colon cancer in 76 patients with colon cancer. The expression level of *miR-449a* was similar in colon cancer tissue and normal colon tissue (Fig. 5a). We next assessed the association of pathological findings and expression level of *miR-449a* in colon cancer tissue. Higher expression of *miR-449a* was correlated positively with depth, differentiation, and size of colon cancer, but was not correlated with lymphatic invasion, venous invasion, or lymph node metastasis (Fig. 5b). We also compared the expression level of *miR-449a* and the prognosis of patients with colon cancer. Overall survival was not affected by *miR-449a* expression; however, disease-free survival was much longer in patients with higher *miR-449a* expression (Fig. 5c). Taken together, these results strongly suggested that there was a negative correlation between *miR-449a* expression and the severity of human colon cancer.

### Expression of Mlh1 was positively correlated with miR-449a

**Figure 6:**
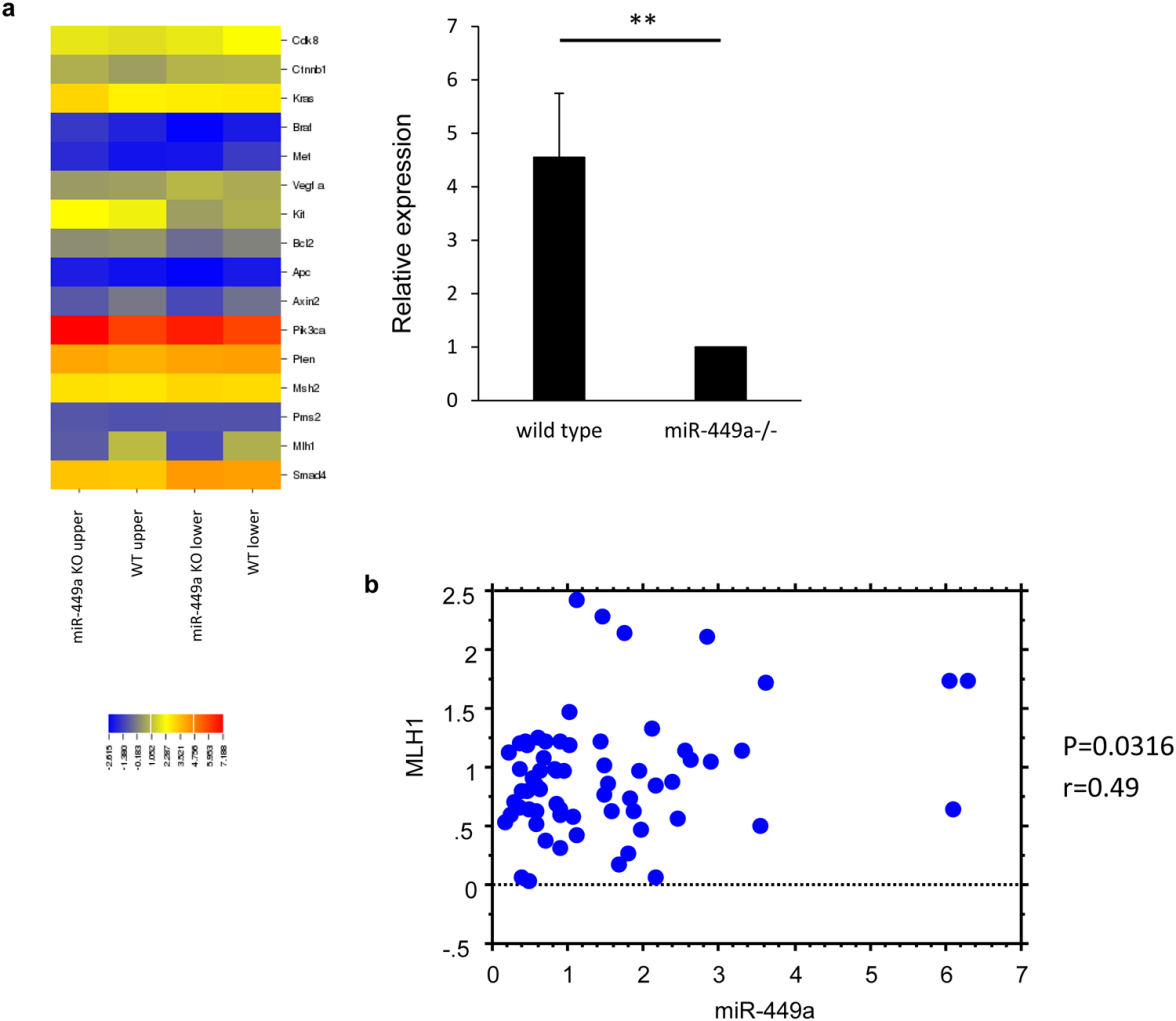
Expression of *miR-449a* was correlated with MLH1 in patients with colon cancer. (**a**) Total RNA was isolated from the upper and lower colons of wild-type and *miR-449a* ^−/−^ mice, and mRNA expression was evaluated by DNA microarray. The expression of *Mlh1* in upper and lower colons from wild-type and *miR-449a* ^−/−^ mice was evaluated by real-time PCR. The data are shown as means ± SDs. **p < 0.01. (**b**) Expression of *miR-449a* and *MLH1* was compared in cancerous tissues from 72 patients with colon cancer. *P* values are shown for each comparison.

Next, we compared the mRNA expression in the upper and lower colons of wild-type and *miR-449a* ^−/−^ mice to identify *miR-449a* target genes relevant to colon tumorigenesis (Fig. 6a). Among 16 known genes associated with colon cancer, *Mlh1* was downregulated in both the upper and lower colons in *miR-449a* ^−/−^ mice compared with those from wild-type mice. The downregulation of *Mlh1* was confirmed by real-time PCR (Fig. 6a). Another 15 genes were not significantly altered by deleting *miR-449a*.

MLH1 is a known tumor suppressor in colon cancer. Therefore, we evaluated whether there was an association between *MLH1* and *miR-449a* expression in patients with colon cancer. The expression of *MLH1* was positively correlated with *miR-449a* expression in 72 patients with colon cancer (Fig. 6b), suggesting that *miR-449a* played a role in susceptibility to colon cancer through controlling *MLH1* expression.

## Discussion

In this study, we searched for miRNAs that were regulated by Notch signaling and found that *miR-449a* was indirectly upregulated by Notch signaling. Although Notch signaling is involved in the development or differentiation of various immune cells^9^, *miR-449a* ^−/−^ mice do not show any defects in immune cell development^27, 28^, a process regulated by Notch signaling. Notably, *miR-449a* ^−/−^ mice showed higher susceptibility to AOM/DSS-induced colon tumorigenesis than wild-type mice, and the expression level of *miR-449a* was inversely correlated with disease severity, including disease-free survival, in patients with colon cancer. Moreover, *miR-449a*-deficient cells expressed lower levels of *Mlh1* than control mice, and the expression of *miR-449a* was positively correlated with *MLH1* in cancerous tissues from patients with colon cancer. These data suggested that *miR-449a* functioned to suppress colon tumorigenesis, at least partly through regulating *MLH1* expression, and highlighted *miR-449a* as a therapeutic target and prognostic marker in the treatment of colon cancer.

The expression of *miR-449a* is frequently decreased in malignant tumors, including gastric and bladder cancer^17, 18^. In these cancers, *miR-449a* may inhibit cell growth or induce senescence and apoptosis by activating the p53 pathway. One recent paper reported that *miR-449a* was downregulated, while STAB2 expression was upregulated in patients with colorectal cancer^29^. However, it is still unclear whether *miR-449a* is directly associated with tumorigenesis of various types of cancer because of a lack of data from *in vivo* models; accordingly, we have established *miR-449a*-deficient mice. In these mice, AOM/DSS treatment increased the incidence of colon cancer and the rate of Ki-67-positive intestinal epithelial cells compared with those in control mice, directly demonstrating that *miR-449a* suppressed colon tumorigenesis and intestinal epithelial cell proliferation. Additionally, our microarray analysis showed that *Mlh1* expression was lower in *miR-449a* ^−/−^ mice than in wild-type mice. MLH1 forms a heterodimer with PMS2 and functions to correct small errors involving mispaired nucleotides during DNA replication^30^. *MLH1* is frequently mutated in patients with colon cancer^30–33^. Furthermore, *Mlh1* deficiency in mice also accelerates colon carcinogenesis when combined with inflammation^34^. Notably, *miR-449a* expression was positively correlated with *MLH1* in patients with colon cancer in our present study. Thus, although it was unclear how *miR-449a* affected *Mlh1* expression, our data suggested that *miR-449a*-mediated upregulation of MLH1 regulated the initiation or progression of colon cancer. The associations of *miR-449a* and MLH1 in terms of tumorigenesis should be analyzed by overexpressing Mlh1 in *miR-449a*-deficient mice, and it is also essential to assess how *miR-449a* regulates MLH1 expression. In addition, the *miR-449* cluster contains sequences and secondary structures similar to those of the *miR-34* family, which was found to be a p53-responsive gene cluster^35, 36^. *miR-34* targets the histone deacetylase SIRT1^37^, leading to the accumulation of acetylated and therefore highly active p53. Moreover, *miR-34* also downregulates several cyclin-dependent kinases, cyclins, and E2Fs^38, 39^, leading to cell cycle arrest. Therefore, the *miR-449* cluster, including *miR-449a*, may undergo similar regulatory processes, contributing to the suppression of colon tumorigenesis by *miR-449a*.

Previous papers have reported that overexpression of *miR-449a* reduced Notch signaling^40^ and that blocking of *miR-449*-binding sites of endogenous human Notch1 or frog Dll1 strongly repressed multiciliogenesis^41^. Furthermore, *miR-449a* reduces cancer cell survival by directly downregulating Notch1^42^. These data indicated that *miR-449a* could suppress Notch signaling. However, our results demonstrated that stimulation of T cell hybridomas with overexpression of the intracellular domain of Notch1 upregulated *miR-449a*. These data were supported by low *miR-449a* expression in Rbpj-deficient T cells. However, we did not detect any defects in the development of T cells, marginal zone B cells, or splenic CD8α^−^ dendritic cells, all of which are regulated by Notch signaling, in *miR-449a* ^−/−^ mice. Therefore, *miR-449a* was not involved in Notch-mediated immune cell development. Notch signaling has also been implicated in tumorigenesis in many cancers, including colon cancer, due to the induction of prosurvival signaling in colonic epithelial cells^43^. Therefore, given the upregulation of *miR-449a* by Notch, Notch-mediated *miR-449a* expression in the colon may function as a self-guarding signal to suppress Notch-induced expression of tumorigenesis genes in the same cells.

In summary, our results revealed that the expression of *miR-449a* was inversely correlated with disease-free survival in colon cancer, and deficiency of *miR-449a* in mice increased susceptibility to AOM/DSS-induced colon cancer. In a previous study, *miR-449a* expression in carcinoma tissues was found to be inversely correlated with the levels of serum carcinoembryonic antigen^18^, supporting our present findings. With the goal of developing novel therapeutic strategies, stimulators of *miR-449a* may have beneficial effects on suppressing colon cancer and identification of target genes for *miR-449a* may yield novel target molecules to suppress colon cancer. Our present data also highlighted *miR-449a* not only as a therapeutic target of colon cancer but also as a prognosis marker. Furthermore, previous papers have demonstrated that the expression of *miR-449a* is associated with progression of lung, gastric, and bladder cancers; thus, our *miR-449a*-deficient mouse model may be a useful tool to address the contribution of *miR-449a* to tumorigenesis in these cancers.

## Methods

### Mice

Six- to 8-week-old C57BL/6 mice were purchased from Japan SLC (Hamamatsu, Japan). Rbpj^flox/flox^ crossed with CD4-Cre transgenic mice were previously reported^13, 44^. All mice were maintained under specific pathogen-free conditions in the animal facilities at Tokushima University, and all animal experiments were approved by the animal research committee of Tokushima University and performed in accordance with our institution’s guidelines for animal care and use.

### Establishment of *miR-449a*^−/−^ mice

Murine genomic DNA of *miR-449a* was cloned by PCR. A 377b genomic *miR-449a* (restriction enzyme: NotI, EcoRV) fragment was replaced with a *neo* resistance gene cassette. E14 embryonic stem cells (1 × 10^7^) were electroporated with 20 μg of linearized targeting vector. G418-resistant colonies were obtained after 10 days. PCR screening for homologous recombination was carried out with a primer specific for the *neo* resistance gene (5′-TATCAGGACATAGCGTTGGC-3′) and an outside primer specific for *miR-449a* (5′-CTGTTCCGTGAATCAAACGG-3′) upstream of the construct. Homologous recombination was subsequently confirmed by BclI digestion of genomic DNA and hybridization with specific probes. Germline transmission of the *miR-449a* mutation was confirmed by Southern blot analysis.

### Flow cytometry

Cells from the thymus and single-cell suspensions from the spleen or lymph nodes were stained with combinations of following antibodies: anti-mouse CD8α (53–6.7), anti-CD44 (IM7), anti-CD62L (MEL-14), anti-TCRβ (H57-597), anti-CD11b (M1/70), and anti-Gr1 (RB6-8C5), from Tonbo Biosciences (San Diego, CA, USA); anti-CD4 (GK1.5) and anti-B220 (RA3-6B2) from BD Biosciences (*Franklin Lakes*, NJ, USA); and anti-TCRγδ (GL-3), anti-F4/80 (BM8), anti-CD21 (8D9), and anti-CD23 (B3B4) from eBiosciences (San Diego, CA, USA). All samples were resuspended in phosphate-buffered saline (PBS) staining buffer containing 2% fetal bovine serum and 0.01% NaN_3_ and pre-incubated for 15 min at 4 °C with 2.4G2 supernatant to block the Fc receptor. Samples were then washed and stained with specific mAbs for 20 min at 4 °C. Data were collected on a FACSCanto II (BD Biosciences) and analyzed using FACS Diva (BD Biosciences) or FlowJo (Tree Star, OR, USA) software.

### Purification of T cells

Total T cells from the spleens of C56BL/6 and *Rbpj* ^flox/flox^ mice crossed with CD4-Cre transgenic mice were purified with a T-cell isolation kit (Miltenyi Biotec, Gladbach, Germany). Total RNA was isolated with RNeasy (Qiagen, Hilden, Germany).

### Microarray

The intracellular domain of mouse Notch1 was cloned into the pKD004 retrovirus vector encoding green fluorescent protein (GFP)^45^. The vectors were transfected with Plat-E cells^46^ with GeneJuice (Merck Millipore, Darmstadt, Germany), and supernatants were collected 2 days after transfection. DO.11.10 cells were infected with retrovirus by centrifuging cells at 2600 rpm for 90 min. The miRNAs were collected using a High Pure miRNA isolation kit (Roche). One hundred nanograms of miRNA was used for the RNA probe. Microarray analyses were performed on a Mouse miRNA microarray 8 × 15 K miRBase 12.0 (Agilent).

Total RNA was isolated from the colons of mice using a Relia RNA Cell Miniprep System (Promega, Madison, WI, USA), and RNA quality was assessed by analysis with an Agilent 2100 BioAnalyzer. Thirty nanograms of RNA was used for the RNA probe. Probe preparation and microarray analyses were performed on a Whole Mouse Genome OligoDNA microarray kit ver2.0 4 × 44 K (Agilent Technologies). The resulting data were normalized using GeneSpring (Agilent Technologies) software. Genes showing at least 3.0-fold changes in expression (*p* < 0.05) between groups were considered to be differentially expressed.

### AOM/DSS treatment

Mice were injected intraperitoneally with 10 mg/kg AOM (Sigma, St. Louis, MO, USA). Seven days later, 2% dextran sodium sulfate (ICN, MW 5,000 kDa) was given in the drinking water for 7 days. Body weights were measured every day after DSS treatment.

### Histological studies

Mouse colon tissues were fixed in 10% formalin neutral buffer solution (Wako) and then embedded in paraffin. Paraffin-embedded sections were cut to 5 μm thickness and stained with hematoxylin and eosin solution. Paraffin-embedded sections were then stained with anti-Ki67 antibodies (D3B5; Cell Signaling Technology) followed by horseradish peroxidase (HRP)-labeled anti-rabbit antibodies.

### Real-time PCR

Total RNA was extracted using RNeasy Plus Mini Kits (Qiagen, Valencia, CA, USA), and cDNA was synthesized using an Omniscript RT Kit (Qiagen). Gene expression was analyzed by qPCR on a Step-One RT PCR system (Applied Biosystems) using SBYR green incorporation. All genes were normalized to *Hprt*, and relative expression was calculated using the ΔΔCT method. All primer pairs were validated for amplification efficiency. The following primers were used: *Mlh1*, Fwd-5′-GCCGGCCAATGCTATCAAAG-3′, Rev-5′-TTGACGTCCACGTTCTGAGG-3′; *Hprt*, Fwd-5′-AGCCTAAGATGAGCGCAAGT-3′, Rev-5′-TTACTAGGCAGATGGCCACA-3′.

### Chromatin immunoprecipitation

Cells were incubated with 1% formaldehyde for 5 min at 4 °C, and the crosslinked chromatin was then sonicated to shear chromatin fragments (200–1000 bp). The sonicated chromatin was immunoprecipitated with anti-Rbpj antibodies (RbpSUH, Cell Signaling Technology), and the negative control was immunoprecipitated with control antibodies. The immunocomplexes were recovered using Dynabeads Protein G (Invitrogen). After treatment with proteinase K, DNA was purified by phenol/chloroform extraction. Real-time PCR was performed to quantify Rbpj-binding *miR-449a* promoter fragments using the following primers: *miR-449a* (450 bp), forward, 5′-GATGCCTAGGACCTAAGTAC-3′ and reverse, 5′-GCCACATAAACCTCTTCCTC-3′; *miR-449a* (11 kbp), forward, 5′-CAACGGATGTTGACGTGTG-3′ and reverse, 5′-CAGCTAGGCTCCATCTCATA-3′; *Cnot3*, forward, 5′-CAAGACATGGGTAGCATCAA-3′ and reverse, 5′-TGGTTTCTAACCGTCTCAAT-3′; and *Dtx1*, forward, 5′-CACACACCCTCCTGCAGTC-3′ and reverse, 5′-CAGGGAGAGAGTCTCGATGC-3′.

### Tumor samples

Pairs of primary colon tumor tissues and adjacent nontumor tissues were collected from 80 patients recruited from Tokushima University. Detailed background information for each tissue donor, including age, sex, clinical staging, tumor location, and survival times after diagnosis, was collected. The patients were ordered based on *miR-449a* expression levels, and *miR-449a* low and high groups were designated based on the median value for all patients. This study was approved by the Institutional Review Board for Human Subject Research at Tokushima University. All participants provided written informed consent to participate in this study, and the consent procedure was approved by the Institutional Review Board. All methods were performed in accordance with the relevant guidelines and regulations.

### Statistics

The means ± standard deviations (SDs) were calculated for all parameters determined. Statistical significance was evaluated using one-way analysis of variance (ANOVA), followed by Fisher’s protected least significant difference test. *P* values less than 0.05 were considered statistically significant. For all experiments, the significance of differences between groups was calculated using the Mann-Whitney U test for unpaired data. The Kaplan-Meier method and log rank test were used to estimate overall survival. The correlation coefficients were analyzed by standard Pearson correlation analysis.

## Electronic supplementary material

Supplementary Information
