# Supplementary material for: MicroRNA-449a deficiency promotes colon carcinogenesis
Source: Sci Rep. 2017 Sep 6;7:10696. doi: 10.1038/s41598-017-10500-0 (PMC5587792; doi:10.1038/s41598-017-10500-0)
Supplement: Supplementary file 1 — Supplementary Information [file 41598_2017_10500_MOESM1_ESM.pdf]

MicroRNA-449a deficiency promotes colon carcinogenesis

Masanori Niki, Kohei Nakajima, Daichi Ishikawa, Jun Nishida, Chieko Ishifune, Shin-ichi Tsukumo, Mitsuo Shimada, Shinji Nagahiro, Yoshinori Mitamura, Koji Yasutomo

Supplementary Figure 1. Niki *et al.*

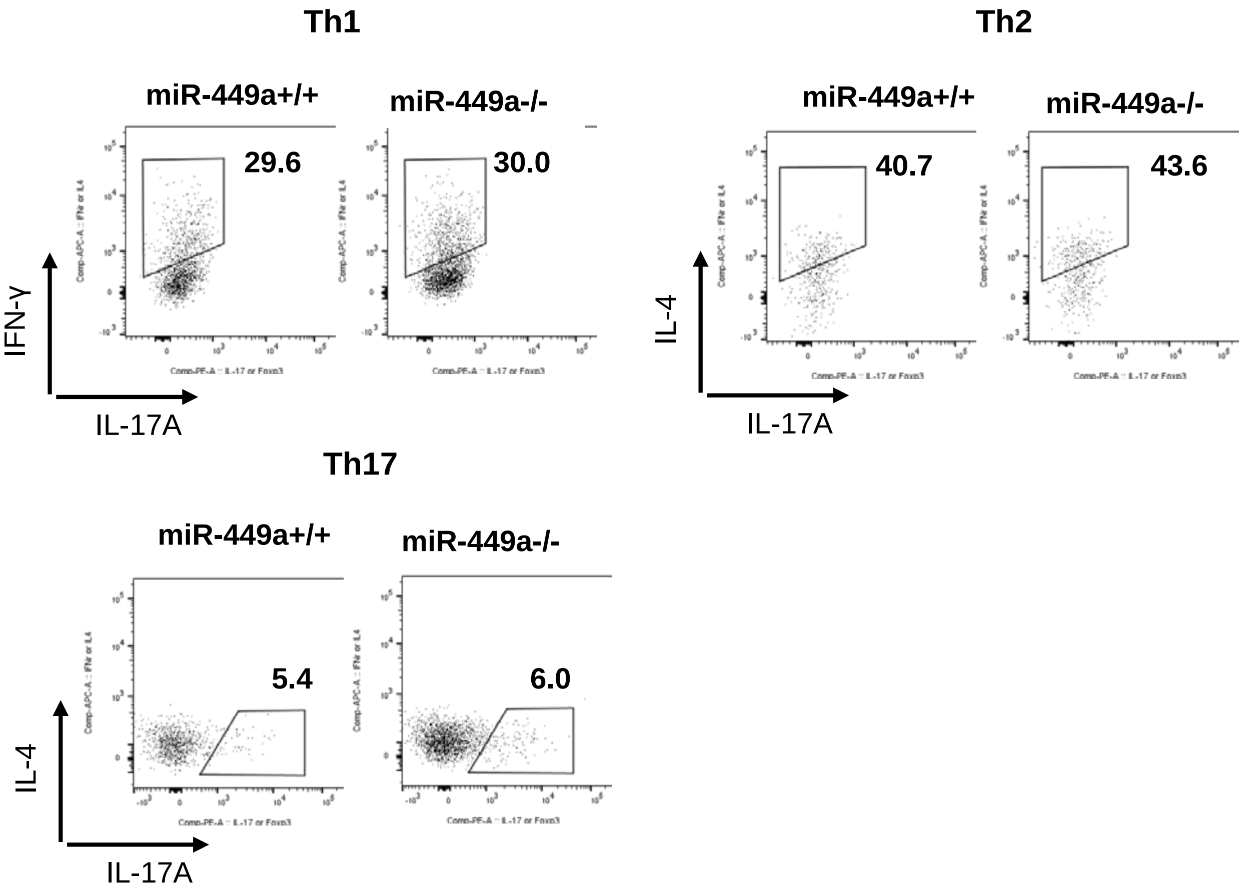

Figure legend

T cells from miR-449a+/+ or miR-449a-/- mice were stimulated with anti-CD3 mAb (1 µg/ml) in the presence of IFN-γ and anti-IL4 mAb (Th1), IL-4 and anti- IFN-γ or TGF-β and IL-6 (Th17) for 3 days, and expression of cytokines were evaluated after stimulation with PMA and ionomycin for 5 hours.

Supplementary Figure 2. Niki *et al.*

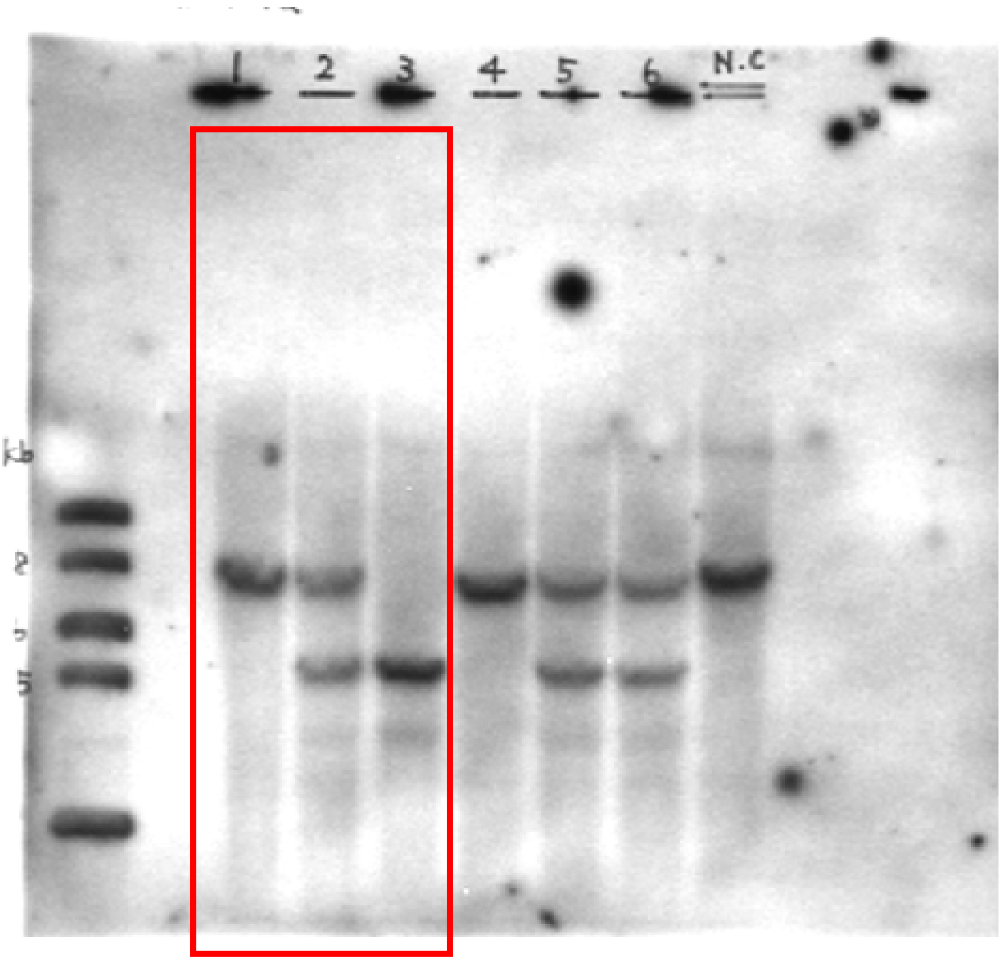

Figure legend

Genomic DNA was digested with Bcl1 and subjected to Southern blot analysis. Genomic DNA from wild-type or *miR-449a*$^{-/-}$ mice was amplified by PCR primers that detected the wild-type or mutant allele. The part in the red rectangle is shown in Figure 2b.
